# The Economic Impact of Herpes Zoster Vaccine Disparities in Elderly United States Blacks

**DOI:** 10.3390/ijerph15102128

**Published:** 2018-09-27

**Authors:** La’Marcus T. Wingate, Keisha Stubbs, Iman Ahmed, Rachel K. Mayaka, Mary K. Maneno, Earl Ettienne, Oluchi Elekwachi, Veronica Clarke-Tasker

**Affiliations:** 1 Center for Minority Health Services Research, 2300 4th Street NW, Annex 3, Washington, DC 20059, USA; rachel.mayaka@bison.howard.edu (R.K.M.); mary.maneno@howard.edu (M.K.M.); earl.ettienne@howard.edu (E.E.); 2 Howard University Research Centers in Minority Institutions, Howard University, 520 W Street NW, Room 436, Washington, DC 20059, USA; 3 Howard University College of Pharmacy, Howard University, 2300 4th Street NW, Chauncey Cooper Hall, Washington, DC 20059, USA; keisha.stubbs@bison.howard.edu (K.S.); iman.ahmed@bison.howard.edu (I.A.); 4 US Food and Drug Administration, Office of Minority Health, 10903 New Hampshire Avenue, Silver Spring, MD 20993, USA; Oluchi.elekwachi@fda.hhs.gov; 5 Howard University College of Nursing and Allied Health Sciences, Howard University, 516 Bryant Street NW, Washington, DC 20059, USA; vclarke-tasker@howard.edu

**Keywords:** herpes zoster, shingles, vaccination, elderly, blacks, seniors

## Abstract

There are persistent disparities with regard to receipt of herpes zoster vaccine among elderly blacks, but no data is available regarding the public health or economic impact of these disparities. A decision tree was constructed with multiple Markov nodes in order to estimate the preventable cases of herpes zoster occurring among elderly blacks due to disparities in receipt of herpes zoster vaccine and to quantify the economic costs associated with these disparities. The model was constructed to examine the number of herpes zoster cases occurring among elderly blacks from the age of 60 to 84 over a 20 year period and also calculated costs due to herpes zoster complications and lost productivity. Achievement of health equity would prevent over 34,500 cases of herpes zoster from occurring in the future and avert over $180 million in lost productivity and treatment costs as a result of these cases of herpes zoster. These results help to show that thousands of cases of herpes zoster could be prevented if blacks were vaccinated at the same frequency as whites and help to show the benefit of implementing viable strategies to achieving this goal.

## 1. Introduction

Herpes zoster (shingles) is a common disease with one million cases occurring annually in the United States, such that 30% of the population is reported to contract the disease during their lifetime [1]. The likelihood of contracting the disease increases with age, ranging from approximately five cases per 1000 people, to 11 cases per 1000 people in those at least 80 [2]. The incidence of disease has been increasing over time and so was four times higher in 2000 to 2007 when compared to 1945 to 1949 [3]. The hallmark symptom of the disease is painful rashes that are normally unilateral by nature [4] and complications involving the cutaneous, visceral, neurological, and ocular symptoms are fairly frequent [5]. The most common complication is post herpetic neuralgia (PHN) which is typified by severe pain that may last three months or more after the resolution of the rashes and may occur in over 10% of cases [5]. 

Herpes zoster imposes a substantial economic burden, particularly in those with disease complications. A recent systematic review indicated that the cost for diagnosing the disease, and simple treatments, can approach $600 for each patient with uncomplicated cases of the disease, yet these costs can exceed $3700 in those with PHN [6]. Likewise, outpatient and consultation costs range from approximately $800 to $1150 in individuals with uncomplicated cases of disease and $1140 to $3560 in patients with PHN [6]. A more recent analysis using advanced techniques demonstrated that the total direct medical costs in those with uncomplicated disease was $1425 and nearly $7300 in those with post herpetic neuralgia [7]. Additionally, data gathered from working age U.S. adults indicate the economic cost to lost productivity to amount to $2350 per person [6,8]. The total economic burden of the disease in the U.S. has been estimated to range from $1 billion to $1.9 billion in the most recently available data, all published before 2010 [9,10,11]. 

Fortunately, two vaccines have been developed to prevent occurrence of herpes zoster [12,13]. The one dose live, attenuated version of the vaccine (ZVL) has the trade name of Zostavax and was approved by the FDA in May of 2006 for use in individuals at least 50 years old, and subsequently recommended for those at least 60 years old by the Advisory Committee on Immunization Practices (ACIP) [14]. This vaccine was demonstrated to be 51% effective in preventing the onset of herpes zoster [15]. The two dose adjuvanted recombinant subunit (RZV) was developed under the trade name Shingrix and was approved by the FDA in 2017 for individuals at least 50 years old and also recommended by ACIP for use in this same age group [13]. This vaccine generally has an effectiveness of at least 90% in the prevention of herpes zoster [16,17]. 

The most robust data regarding uptake of a herpes zoster vaccine is available for ZVL due to the longer time period it has been on market. Initial uptake of the vaccine was low among all seniors, as 7.5% of Caucasians and 2.5% of blacks had received the vaccine by 2008 [18]. Accordingly, Healthy People 2020 targets were set for 30% of eligible seniors to receive the vaccine [19]. Recent surveillance data indicates that the proportion of all seniors at least 60 years old receiving ZVL has nearly doubled from 2011 to 2015, increasing from 15.8% to 30.6% during this time period [7,8,9,10,11]. Regrettably however, the disparity between blacks and whites has increased during this time period such that 34.6% of whites at least 60-year old had received ZVL, in contrast to 13.6% of blacks in this same age group in 2015 [20,21,22,23,24].

The continued failure to achieve health equity between blacks and whites in herpes zoster vaccination status is likely to have substantial economic and public health consequences, especially as the elderly U.S. population increases. In spite of the long standing disparity between blacks and whites with regard to receipt of ZVL, no studies have been conducted to help quantify the public health and economic consequences associated with this disparity. Accordingly, the objectives of this study were to estimate the number of preventable cases of herpes zoster occurring in elderly blacks, due to failure to achieve health equity in ZVL vaccination status and to quantify the economic burden associated with these cases of disease. 

## 2. Materials and Methods

### 2.1. Methods Overview

A decision tree with multiple Markov nodes was constructed using TreeAge Pro© software (2014) (TreeAge Software Inc., Williamstown, MA, USA) to conduct an economic evaluation to determine the amount of preventable cases of herpes zoster occurring in elderly blacks. Using Markov models allowed a simulation to be conducted, whereby seniors could have no herpes zoster or transition to a state where they contracted herpes zoster with the possibility of developing disease related complications. All individuals began the simulation with no herpes zoster and the transitions to other states took place in accordance with epidemiological data. The transitions took place at one-year cycles. The model was constructed in a manner to estimate costs from the health care perspective while also incorporating indirect costs due to lost productivity, as patients incur the relevant costs when they transition to a state of uncomplicated herpes zoster or herpes zoster with complications. The study was submitted to the Howard University Institutional Review Board and conferred an exempt status. 

### 2.2. Description of Population

The population for this study was based upon the elderly Black population found in the United States between the ages of 60 to 84 which numbered over 6.1 million as of 2015 as seen in Table 1 [25]. The vaccination rates with regard to ZVL were derived from the most recently available epidemiological data published using 2015 records [25]. Two separate scenarios were modeled in accordance with the data. In the status quo scenario, 12.7% of blacks between 60 to 64 and 14.1% of blacks 65 to 84 were assumed to have received the vaccine in 2015. For the health equity scenario, the model was constructed so that blacks received ZVL at the same frequency as whites. Therefore, the proportion of blacks receiving ZVL was 25.1% for those age 60 to 64, and 38.3% for those age 65 to 84 in the health equity scenario. In both cohorts, the individuals were followed for a 20 year time period to determine the number of individuals contracting herpes zoster after accounting for background mortality [26]. No additional people were assumed to be vaccinated after the first year of the simulation, as the model was constructed primarily to determine the implications of the current 21% disparity between blacks and whites in receipt of the ZVL vaccine. Maintaining the immunization rates utilized at the onset of the simulation allows for there to be a constant difference between the proportion of blacks receiving vaccination in the status quo and health equity scenarios.

### 2.3. Epidemiological Parameters

The incidence of herpes zoster in the absence of vaccination was modeled using data from a national claims database which contained information for approximately four million United States residents with private insurance [2,27]. As ZVL is not indicated for use in those with compromised immune systems [12], the age specific incidence rates were derived specifically from immunocompetent patients. Accordingly, the age specific incidence rate varied from 5.90 per 1000 person years for those under 70 to 9.76 per 1000 person years for individuals at least 80 [2,27].

The vaccine effectiveness was modeled so that it varied with the age it was received and the number of years since administration. In accordance with clinical trial data and previous economic evaluations, the vaccine effectiveness was derived to have decreasing benefits as the age at administration (a) and time elapsed since vaccination (t) increased [15,27]. The equation used to determine the effectiveness (E) of a vaccine at any given point as a function of the age in which it was initially administered is shown below in Equation (1). 

Individuals developing herpes zoster could experience either uncomplicated disease, or develop one of several complications. The types of complications included in the model were PHN, neurological, cutaneous, ocular, and other states. The frequency of complications among those contracting herpes zoster was derived from data obtained in a clinical trial which collected data on individuals developing herpes zoster without the benefit of a vaccine. The occurrence of complications was more frequent in elderly individuals. The most frequent complication was PHN which occurred in 12% to 32% of individuals based on age.

E_t_(a) = (−0.000541a^2^ + 0.054243a − 0.612759)^−0.083at^ (1)

The direct costs of treating cases of herpes zoster were derived from recent data, which shows the health care costs incurred in the treatment of the disease. The study used data from a large United States secondary claims database in estimating the additional costs incurred for patients with herpes zoster by age after adjusting for demographic and clinical characteristics [7]. The patients with herpes zoster were matched to up to four patients without herpes zoster on the basis of age, gender and type of health plan enrollment. The analysis included cost for a one year period following the initial diagnosis and included costs related to medications, visits to physician’s offices, emergency department visits, and inpatient hospitalizations. Depending upon the age at which the disease developed, patients with uncomplicated herpes zoster had annual costs which were $1210 to $3804 higher than those without the illness. Patients contracting the disease and experiencing PHN had annual health care costs that were $4670 to $11,147 higher than those with no herpes zoster [7]. 

Costs related to complications other than PHN are scarce in the medical literature as noted in a systematic review of costs associated with herpes zoster [6]. Accordingly, the costs for treating herpes zoster complications other than PHN were derived from a retrospective study using population based data collected from 1996 to 2001 [9]. All direct medical costs were updated to 2016 dollars by using the Medical Care component of the Consumer Price Index [28]. The indirect medical costs were abstracted from a previous cost effectiveness evaluation that estimated the lost productivity as a function of the proportion of the elderly population that worked and the corresponding weekly wages [29]. These age specific data were updated to 2016 values using the Social Security Wage Index [30]. 

## 3. Results

### 3.1. Projected Number of Herpes Zoster Cases 

As shown in Table 2, there would be over 610,000 cases of herpes zoster occurring among elderly blacks over the next 20 years if the current ZVL vaccination rates were maintained. The greatest number of cases would occur among those aged 60 to 64, where over 223,000 cases of herpes zoster would be projected to take place. In contrast, under 39,000 cases of disease would be projected to occur among seniors currently between 80 and 84.

In the health equity scenario, just over 576,027 cases of herpes zoster cases would be projected to occur among the cohort if ZVL vaccination rates among elderly blacks were the same as those seen among whites. In this scenario as well, most cases would be seen among the cohort aged 60 to 64 at the outset of the simulation where approximately 214,025 cases would take place. Only 37,650 cases would take place during this time period among the oldest cohort that was between the ages of 80 to 84 at the start of the simulation.

### 3.2. Potential Herpes Zoster Cases Prevented

Overall, achievement of health equity in ZVL vaccination status would prevent over 34,500 cases of herpes zoster from occurring among elderly blacks over the 20 year time period. Most cases of the disease would be prevented in the younger age groups, where more seniors would survive the entire 20-year time period. Over 20,000 of the averted cases would occur in those elderly populations under the age of 70 at the start of the simulation. In contrast, only 1200 cases were prevented in those at least 80 years old, where many individuals would have a shorter life expectancy. 

### 3.3. Projected Costs of Herpes Zoster Cases

The total costs related to treatment and lost productivity in the status quo scenario would exceed $3.3 billion as shown in Table 3. The costs attributable to seniors age 60 to 64 at the outset of the simulation would exceed $1 billion. The costs associated with herpes zoster infection had an inverse relationship with increasing age, decreasing to approximately $256 million in the cohort of seniors between 80 and 84. 

The overall costs related to treatment and lost productivity in the health equity scenario would also represent a substantial economic burden exceeding $3.1 billion as shown in Table 3. The costs ranged from $247 million in seniors in the oldest cohort to $1.0 billion in the elderly blacks age 60 to 64 in the initial year of the simulation. 

### 3.4. Potential Herpes Zoster Associated Costs Prevented

The achievement of health equity in ZVL vaccination status could avert approximately $182 million in costs associated with herpes zoster. The bulk of the potential averted costs would occur in those seniors under 70 where more than $110 million in savings could be realized. However, even among those aged 75 to 79, there could be nearly $22 million in costs prevented related to treatment and lost productivity.

## 4. Discussion

In this study it was demonstrated that over 34,500 cases of herpes zoster could be averted through the realization of health equity, with regard to the receipt of ZVL among the cohort of elderly blacks between the ages of 60 to 84 over a 20-year period. Likewise, achievement of this goal would avert over $182,000 in costs related to treatment of the disease and lost productivity. Our study demonstrated that the greatest impact of these disparities would be realized among those seniors below the age of 70. This is due in part to the fact that the vaccine is less effective in older populations [14]. 

This research helps to underscore the importance of understanding the reasons why so few eligible adults in general have received the vaccine. Disparities have been observed in receipt of the vaccine ever since it appeared on the market [18], and less than 35% of seniors overall have received ZVL [24], indicating that there may be significant barriers to receipt of the vaccine. Notable barriers to health care providers for providing the vaccine include the fact that it has special storage requirements, is costly to procure, and is reimbursed through Medicare Part D with which many providers are not familiar. Accordingly, approximately 40% of physicians had strongly recommended the vaccine to their patients [31]. 

However, emerging evidence has helped to demonstrate that pharmacists may be well positioned to assist in the provision of ZVL as they are familiar with Medicare Part D and are able to accommodate the special storage requirements. Multiple studies illustrate the success of promotional campaigns conducted in community pharmacies that have helped to increase uptake of ZVL [32,33,34,35]. Promotional campaigns like these may be one of the contributing factors in the promising increase seen in the uptake of the vaccine among elderly whites in recent years, as they have already exceeded Healthy People 2020 goals [19]. Indeed, one third of individuals receiving the vaccine cited media or advertisements as one of the venues through which they were made aware of the vaccine [36]. 

One notable feature among many of the promotional campaigns is that there is a scarcity of information regarding the ethnic background of the patients included. When information about the ethnic background of patients is provided, it demonstrates that there is a lack of involvement of minority populations [32,33,34,35]. If ZVL is not promoted to the same extent among minorities, it may be surmised that there will be a lower awareness of the vaccine among minority populations. Research does indeed indicate that there is significantly lower awareness of the vaccine among blacks when compared to whites [36]. This may allude to the possibility that although there may be sufficient promotion of the vaccine in some cases, it may not be reaching minority patients in an adequate manner, either due to lack of cultural sensitivity or general absence of promotions among these populations. 

This study focused on ZVL as there is currently a lack of information regarding any type of differential uptake of RZV among whites or blacks. The RZV vaccine is over 90% effective. Hence, if there are any differences demonstrated with receipt of this vaccine, there would be a lower number needed to treat in order to realize a benefit from the vaccine and in turn, from potential cases of herpes zoster that could be averted and any associated costs related to treatment and lost productivity [12]. This only helps to show that more urgency is needed in helping to ameliorate the disparities seen so far regarding the receipt of the vaccine as effective strategies exist, but they have not been sufficiently employed in reaching minorities. 

This economic evaluation demonstrated not only the potential cases of herpes zoster that could be averted with achievement of health equity, but also the potential costs associated with these preventable cases of disease. This is notable because of the potential impact that contracting shingles may have on elderly individuals with a fixed income, particularly blacks. It is well known that medical bills can impose financial hardship on individuals. One study indicated that 52% of blacks between the ages of 19 to 64 indicated that they had problems with medical bills [37]. This was noticeably higher than the proportion of whites (28%) indicating that they had such a problem. However, what may be more concerning is the fact that this was also higher than the figure of 43% of sicker individuals with a chronic condition, or no better than fair health, who stated they had a problem with medical bills. When looking at elderly individuals, blacks also tend to be more vulnerable to financial hardship as relates it to medical bills. A national study of elderly individuals demonstrated that blacks had 2.6 times greater odds of having medical debt when compared to whites [38].

This study has some limitations. One limitation is that there is no viable way to estimate how many of those currently unvaccinated seniors will obtain vaccinations in the future. However, the primary emphasis of the study was to highlight the impact due to the differences seen in receipt of the vaccine, and this was adequately fulfilled with the current study design. In the case that differences between blacks and whites continue to increase, the estimates cited here regarding the impact of disparities are actually conservative and underestimate the true impact of these differences. Another potential weakness of the study is the fact that we are unable to tell exactly how much of the disease related costs are borne by the patients. We are able to tell that these preventable cases of herpes zoster exacerbate the financial well-being of a population which is financially vulnerable but we are not able to tell to what extent. Inclusion of costs for lost productivity may not be as intuitive as including costs related to treatment of the disease. However, inclusion of productivity costs is recommended in economic evaluations, and in some cases productivity costs are included for non-working individuals to quantify the lost economic productivity [39,40]. In this study, the method employed only accounted for productivity costs among working seniors. Only a small portion of seniors over age 65 work, but the percentage doing so is increasing over time. In 1992, approximately 18% of seniors between 65 and 74 worked, however this is projected to increase to over 30% by 2022 [41]. Accordingly, this study actually underestimates the full economic burden associated with preventable cases of herpes zoster. 

Future studies could build upon the present study in several ways. Once reliable data become available regarding seniors receiving RZV, similar methodologies could be employed to determine the impact of disparities in receipt of this vaccine on health and economic outcomes between whites and blacks. Hispanics also have persistent differences with regard to the use of vaccines to prevent herpes zoster, and future studies could explore the impact of disparities in this population as well. Additionally, future studies could also employ more precise economic methods to allow for an estimate of how much of the health care expenses are borne by seniors and also find ways to value any lost productivity for nonworking seniors. 

## 5. Conclusions

This study was able to find that tens of thousands of cases of herpes zoster could be prevented if elderly blacks received available vaccines at the same frequency as whites. In addition, hundreds of millions of dollars could be saved relating to herpes zoster treatment and productivity costs among these elderly blacks as well. This data helps to emphasize the importance of finding effective strategies to increase the rates of vaccination among the elderly black population. 

## Figures and Tables

**Table 1:** Epidemiological and cost inputs for decision analysis models.

Parameter	Value	Source
Number of Black Non-Hispanics in U.S.		[25]
60 to 64	2,191,197	
65 to 69	1,642,788	
70 to 74	1,082,966	
75 to 79	749,209	
80 to 84	493,406	
Vaccine coverage under status quo		[20]
60 to 64	12.7%	
At least 65	14.1%	
Vaccine coverage under health equity		[20]
60 to 64	25.1%	
At least 65	38.3%	
Incidence of herpes zoster per 1000-person years		[2,27]
60 to 69	5.90	
70 to 79	7.76	
At least 80	9.76	
Initial vaccine effectiveness		[15,27]
60	69.4%	
65	62.7%	
70	53.3%	
75	41.2%	
Background mortality rate per 100,000		[26]
60 to 64	1571.1	
65 to 69	2069.7	
70 to 74	2694.9	
75 to 79	4311.4	
80 to 84	6427.4	
At least 85	12,364.4	
Direct costs for uncomplicated herpes zoster		[7,28]
60 to 64	$1510	
65 to 69	$1628	
70 to 79	$2153	
80 and over	$2877	
Direct costs for herpes zoster with PHN		[7,28]
60 to 64	$6680	
65 to 69	$7027	
70 to 79	$9311	
80 and over	$12,142	
Direct costs for patients with complications		[9,28]
Ocular	$4275	
Neurologic	$10,136	
Skin	$10,137	
Other	$10,712	
Indirect costs for patients with herpes zoster		[9,30]
60 to 64	$2434	
65 to 69	$936	
70 to 74	$552	
Over 75	$233	

Notes: PHN = post herpetic neuralgia; U.S. = United States.

**Table 2:** Potential herpes zoster cases prevented in elderly blacks by achieving health equity in shingles vaccine status.

Age Group	Cases Under Status Quo	Cases under Health Equity	Cases Prevented with Health Equity
60 to 64	223,655	214,025	9630
65 to 69	171,742	158,762	12,980
70 to 74	108,669	101,407	7262
75 to 79	67,675	64,182	3494
80 to 84	38,851	37,651	1200
Total	610,592	576,027	34,566

**Table 3:** Potential herpes zoster related costs saved among elderly blacks by achieving health equity in shingles vaccine status.

Age Group	Costs under Status Quo	Costs under Health Equity	Costs Prevented with Health Equity
60 to 64	$1,063,236,492	$1,019,136,023	$44,100,469
65 to 69	$926,786,407	$859,866,288	$66,920,119
70 to 74	$639,713,214	$598,356,151	$41,357,064
75 to 79	$429,627,308	$407,649,262	$21,978,046
80 to 84	$255,917,357	$247,921,861	$7,995,496
Total	$3,315,280,778	$3,132,929,585	$182,351,194
